# Social isolation and aggression training lead to escalated aggression and hypothalamus-pituitary-gonad axis hyperfunction in mice

**DOI:** 10.1038/s41386-024-01808-3

**Published:** 2024-02-09

**Authors:** Vinícius Elias de Moura Oliveira, Florence Evrard, Melanie C. Faure, Julie Bakker

**Affiliations:** 1 Laboratory of Neuroendocrinology, GIGA-Neurosciences, University of Liege, 4000 Liege, Belgium; 2 Institute of Pathophysiology, University Medical Center of the Johannes Gutenberg University Mainz, Duesbergweg 6, 55128 Mainz, Germany

**Keywords:** Emotion, Social neuroscience, Neurophysiology, Psychiatric disorders

## Abstract

Although the participation of sex hormones and sex hormone-responsive neurons in aggressive behavior has been extensively studied, the role of other systems within the hypothalamus-pituitary-gonadal (HPG) axis remains elusive. Here we assessed how the gonadotropin-releasing hormone (GnRH) and kisspeptin systems are impacted by escalated aggression in male mice. We used a combination of social isolation and aggression training (IST) to exacerbate mice’s aggressive behavior. Next, low-aggressive (group-housed, GH) and highly aggressive (IST) mice were compared regarding neuronal activity in the target populations and hormonal levels, using immunohistochemistry and ELISA, respectively. Finally, we used pharmacological and viral approaches to manipulate neuropeptide signaling and expression, subsequently evaluating its effects on behavior. IST mice exhibited enhanced aggressive behavior compared to GH controls, which was accompanied by elevated neuronal activity in GnRH neurons and arcuate nucleus kisspeptin neurons. Remarkably, IST mice presented an increased number of kisspeptin neurons in the anteroventral periventricular nucleus (AVPV). In addition, IST mice exhibited elevated levels of luteinizing hormone (LH) in serum. Accordingly, activation and blockade of GnRH receptors (GnRHR) exacerbated and reduced aggression, respectively. Surprisingly, kisspeptin had intricate effects on aggression, i.e., viral ablation of AVPV-kisspeptin neurons impaired the training-induced rise in aggressive behavior whereas kisspeptin itself strongly reduced aggression in IST mice. Our results indicate that IST enhances aggressive behavior in male mice by exacerbating HPG-axis activity. Particularly, increased GnRH neuron activity and GnRHR signaling were found to underlie aggression whereas the relationship with kisspeptin remains puzzling.

## Introduction

Aggressive behavior is an innate behavior displayed by animals in order to get access to important resources for their survival [1, 2]. Apart from its ecological role, pathological and out-of-context aggression constitutes a core symptom of several psychiatric disorders such as conduct and antisocial personality disorders, schizophrenia, major depression, and post-traumatic stress disorder [3–7].

The fact that violence generates a severe socioeconomic burden on society [3, 6] has led scientists to develop preclinical animal models [4, 5, 7] to understand the neural underpinnings of escalated aggression. Among those models, social isolation and engaging in successive aggressive experiences (aggression training) were found to entail robust effects on inducing exaggerated aggression [7, 8]. Additionally, both conditions are known to disrupt social behaviors in humans by triggering psychological distress and violent behaviors [3, 9–11].

Regarding neurobiological factors, attention has been given to several systems such as neuropeptides [4, 8, 12, 13], hypothalamic-pituitary-adrenal (HPA) axis [4, 7], and sex hormones [8, 14]. Specifically, the role of sex hormones [8, 14–19] and sex hormone-responsive neurons [8, 20–23] has been extensively studied in the context of aggression. Yet, the participation of other brain hubs within the hypothalamus-pituitary-gonadal (HPG) axis which regulates sex steroid production and release remains largely unknown.

Gonadotropin-releasing hormone (GnRH) neurons are classically found in the rostral preoptic area (rPOA), medial septum (MS), and anterior hypothalamus. Those neurons are the main node of reproduction in animals [24] as they act on the gonadotrophs in the anterior pituitary to trigger luteinizing hormone (LH) and follicle-stimulating hormone release into the circulation. Those glycoproteins will act at the level of the gonads to stimulate sex steroid production and release to enable reproduction [24–26]. Thus, since aggressive behavior itself [27, 28] as well as successive aggressive interactions and social isolation are known to increase plasma testosterone [8, 14, 17, 18, 27, 28] and to some extent LH [27, 29] (main hormonal output of GnRH neurons), one could hypothesize that GnRH neurons are recruited during aggressive interactions and might be impacted by the social context.

Further evidence of the participation of GnRH in aggressive behavior comes from the finding that either immunocastration using vaccines targeting GnRH or chronic agonism of GnRH receptors (GnRHR), aiming to desensitize the HPG axis, reduce aggression in male pigs [30] and Macaques [31], respectively. Similar effects have been described in a male patient with autism spectrum disorder and high aggression [32]. Importantly, GnRHRs are found in brain regions classically known to modulate aggression such as the lateral septum (LS), ventromedial hypothalamus (VMH), and the cortical amygdala (CoA) [33].

Of note, GnRH neuronal activity is regulated mainly by two neuropeptidergic populations: kisspeptin neurons in the anteroventral periventricular nucleus of the hypothalamus (AVPV) and **K**isspeptin-**N**eurokinin B (NKB)-**D**ynorphin (**KND**y) neurons in the arcuate nucleus of the hypothalamus (ARC) [24, 34–37]. Whereas the role of kisspeptin in male social behaviors remains understudied, the blockade of neurokinin receptor 3 (NK3R), which is co-expressed by KNDy neurons [37, 38], was shown to decrease aggression in highly aggressive socially isolated male mice [39].

Here we used a combination of social isolation and aggression training (IST) [12] to induce escalated aggression in male mice in order to evaluate its effects on GnRH, kisspeptin, and KNDy neuron activity as well as HPG-axis function. Additionally, we manipulated peptide signaling and expression via pharmacological and viral tools to assess its impact on aggressive and social behaviors.

## Methods

### Ethical statement

All procedures described here were approved by the local Animal Ethics Committee of the University of Liège (protocol number 22-2469).

### Animals

Typically, experiments were carried out in adult (8–12 weeks) C57BL6/J male mice, which were purchased from Charles River Laboratories (Les Oncins, France). In particular, neuronal ablation experiments were conducted in Kiss::cre or WT mice (6–12 months) bred in the animal facilities of the University of Liège. For aggressive behavior experiments, male CD-1 mice (5–6 weeks) were used as intruders whereas for sexual behavior receptive (estrous) females C57BL6/J (6–8 weeks) were used. All stimulus animals were obtained via Charles Rivers Laboratories (Les Oncins, France). Subjects were kept under controlled laboratory conditions in ventilated shelves with (12:12 h light/dark cycle; lights off at 10:30 h, 21 ± 1 °C, 60 ± 5% humidity, standard mouse nutrition (RM3) and water ad libitum). Stimulus and experimental animals were housed in large mouse cages (42 × 32 × 18 cm) with sawdust bedding in groups of four. Importantly, during the experimental phase, mice were singly housed in individual mouse cages (33x16x13cm) in order to enhance their aggressive behavior.

### Resident intruder test (RI)

The RI took place in the early dark phase under dim red light conditions. Male mice were confronted with an unfamiliar same-sex intruder for 10 min. Intruders weighed between 10 and 20% less than residents [8, 40, 41]. Behavior was scored live and posteriorly via videos by a blind observer using JWatcher event recorder Program [12, 42]. As previously described [12] the percentage of time spent on four major sets of behaviors was scored: (i) aggressive behavior, consisting of attacks, threats, chases, tail rattles, offensive grooming, offensive up-right; (ii) neutral behaviors, consisting of exploring (investigating the home-cage), drinking and eating, autogrooming, immobility; (iii) social behaviors (non-aggressive social interactions, sniffing, following); and (iv) defensive behavior (submissive posture, kicking a pursuing intruder with hind limb). In addition, we scored the frequency of attacks as well as the latency to the first attack.

### Sexual behavior

Sexual behavior was conducted as previously described [43]. For detailed information please see supplementary information.

### Experimental design

#### The social isolation and aggression training protocol (IST)

**Fig. 1:**
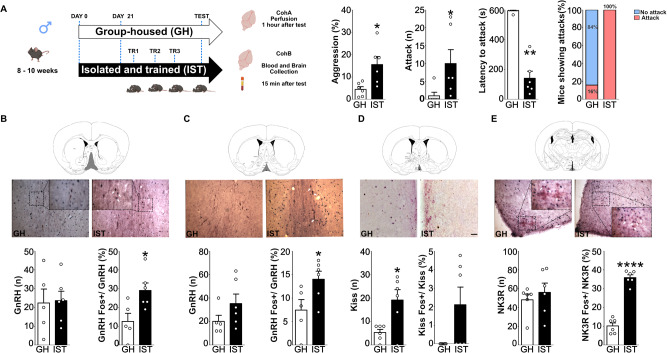
Social isolation and aggression training exacerbate aggression in male mice and increase the activity of GnRH and Kisspeptin neurons. Eight to ten weeks-old male mice were either kept housed in groups (GH) or single-housed and underwent 4 consecutive resident intruder (RI) tests (TR: training). Subjects from both conditions were then split into two cohorts: Cohort A (CohA) was used for neuronal activity experiments whereas Cohort B (CohB) was used for blood collection and hormonal assays (**A**). Isolated and trained (IST, black bars) mice displayed a higher percentage of time on aggression (two-tailed Student’s *t* test t_(10)_ = 2.93, *p* = 0.01), a higher number of attacks (Mann–Whitney U test U = 3.0, *p* = 0.01), and shorter attack latency (U = 0.0, *p* = 0.002) when compared to GH controls (white bars). Furthermore, all IST animals attacked the intruder compared to 16% of the GH mice (Fisher exact test, *p* < 0.00001) (**A**). Regarding neuronal activity, IST mice showed an increased percentage of GnRH neurons co-expressing cFOS in the medial septum (t_(9)_ = 2.8, *p* = 0.02) (MS, **B**) and in the rostral preoptic area (t_(9)_ = 2.4, *p* = 0.04) (rPOA, **C**). IST mice also exhibited a higher number of anteroventral periventricular (AVPV) kisspeptin neurons (t_(10)_ = 3.0, *p* = 0.01) (**D**) and a higher percentage of colocalization between neurokinin-3 receptor (KNDy neuron marker) and cFOS (t_(10)_ = 11.7, *p* < 0.0001) (**E**). Apart from AVPV-Kisspeptin, no other neuronal numbers were changed between GH and IST mice (MS GnRH): t_(9)_ = 0.1, *p* = 0.89; RPOA GnRH: t_(9)_ = 1.5, *p* = 0.17; NK3R KNDy: t_(10)_ = 0.6, *p* = 0.5; AVPV Kiss-Fos: U = 9, *p* = 0.2. *p < 0.05; **p < 0.01;****p < .0001 vs GH. Data are presented as mean + s.e.m. Scale bar 100 μm.

The social isolation and aggression training (IST) protocol was adapted from the procedure developed by Oliveira and co-authors in female rats, which has been shown to induce robust effects on exacerbating aggressive behavior [8, 12, 44, 45]. Therefore, mice from all different strains used to perform this study were assigned to either the IST (high-aggression) or group-housed (GH, control, low-aggression) condition. IST subjects were kept single-housed for 3 weeks in individual cages and were subsequently exposed to 3 consecutive, 10-minute-long RIs, daily. Group-housed animals were kept in groups of 4 animals per cage and were single-housed 2 h prior to lights off, and subsequently tested in the RI (Fig. 1A). Importantly, in pharmacological experiments targeting the IST condition, levels of aggression between vehicle and drug groups were counterbalanced and randomized using the live scoring data of the previous training sessions, to make sure groups did not differ in terms of the total percentage of time spent on aggression, total number of attacks or attack latency before treatments (not shown). Finally, no crossover effects were found when comparing vehicle groups from different treatment days for both housing conditions.

#### Endogenous measurements

Mice were split into two cohorts: cohort A (neuronal activity experiments; *n* = 6) and cohort B (hormonal measurements *n* = 8) (Fig. 1A). After either being assigned to the IST or the GH condition animals underwent the protocols as described above (Fig. 1A). Animals of *Cohort A* were then exposed to a 10-min RI and left undisturbed in their home cage for 1 h. Next, animals were deeply anesthetized with a solution of Euthasol (pentobarbital sodium, 200 mg/Kg) and transcardially perfused using phosphate buffer (PBS, 0.1 M) followed by paraformaldehyde (PFA, 4%). Brains were postfixed overnight in PFA 4% followed by dehydration in sucrose (30%) for 2–3 days and snap-frozen in dry-ice and isopentane (Sigma-Aldrich, PHR166). Mice of *Cohort B* either were kept GH or underwent the IST protocol as described above. On the test day, those animals were confronted with an intruder (10 min), deeply anesthetized with Euthasol 10 min after the test, decapitated, and had their trunk blood collected. Brains were harvested, postfixed in PFA 4%, dehydrated in sucrose, and snap-frozen. Blood was stored in ice, centrifuged (4°, 1500 G, 15 min), and serum was stored at −20 °C until hormonal assays took place.

#### Experiment A

C57BL6/J mice underwent the IST protocol. After the third training session, on day 25 animals were injected intraperitoneally (i.p.) either with deslorelin (GnRH receptor agonist) or vehicle and tested on the RI 30 min after injection. After a one-day (day 27) washout (WO: no RI or i.p. injection) mice were tested again following a deslorelin or vehicle injection in a cross-over (within-subjects) design. Three days later (WO) subjects were then injected with either kisspeptin-10 or vehicle i.p. 2 h before the RI. On day 32 (after 1-day WO) animals were again injected either with kisspeptin-10 or vehicle (i.p.) in a crossover (within-subjects) design. Finally on day 35, IST mice were either injected with vehicle or cetrorelix (GnRH receptor antagonist) 40 min before the RI in a between-subjects design (Figs. 4A and 5A.

#### Experiment B

As deslorelin enhanced aggression in IST mice (Fig. 4), we decided to test whether GnRHR agonism alone could enhance the mild levels of aggression displayed by GH mice. Thus, GH mice were also injected either with deslorelin or vehicle 30 min before the RI. After the experiment, animals returned to their original groups and left undisturbed for 1 day (WO). On day 3, GH mice were again single-housed (see above) and either injected with deslorelin or vehicle in a within-subjects design (Fig. 4A).

#### Experiment C

Similarly to Experiment A, mice underwent the IST protocol. On day 25, IST mice were either injected with senktide i.p. (NK3R agonist) or vehicle, 30 min prior to the RI. Following a 1-day washout, animals were again injected with sektide or vehicle in a cross-over within subjects design. On day 30 (after a 3 days WO), IST mice were then injected with osanetant i.p. (NK3R antagonist) 30 min before the RI in a between-subjects design (Fig. 3A).

#### Experiment D

Kiss::cre or WT mice underwent stereotaxic surgery and were injected into the AVPV with AAV1-flex-taCasp3-TEVp (6.94 × 10^11^ genomic copies per ml) in order to specifically ablate AVPV-kisspeptin neurons [22, 46]. After 3 weeks (day 21), mice started the training phase of the IST protocol, by going through the RI for 3 consecutive days. We chose this approach to evaluate whether ablation of kisspeptin neurons affected baseline (without training) aggressive behavior or only training-induced aggression, as we found that IST animals exhibited higher numbers of kisspeptin neurons in the AVPV. Three days after the third RI (RI3) animals were allowed to interact with estrous/receptive females for 20-min. After a resting of 4 days, animals were again tested for sexual behavior (Sex 2, Fig. 5A) and subsequently perfused to confirm viral ablation. WT mice were also compared with non-surgery animals (not shown, *n* = 3) as there were no behavioral or neuronal number differences between non-surgery and WT those animals were pulled as the control group (Ctrl).

### Stereotaxic surgery

Stereotaxic surgery was performed under semi-sterile conditions and according to the procedures described in the animal license [46]. For details please see supplementary information.

### Drugs

For information regarding doses and time points please see supplementary information.

### Immunohistochemistry

After perfusion, brains were cryo-cutted and slices (40 µm) containing the target regions were collected in cryoprotectant solution and stored at −20 °C until the experiment took place. A series of 6–8 slices were used for immunostaining. For neuronal activity, slices were stained using the immunoperoxidase substrate method whereas for confirming cell ablation slices were stained using immunofluorescence (for protocol details please see supplementary information).

### Sex steroid assays

Testosterone (Demeditec, DEV9911, detection limit 0.024 ng/ml) and Progesterone (Demeditec, DEV9988, detection limit 0.0156 ng/ml) were measured via commercial ELISA kits following the specifics of those kits.

### LH assay

LH was measured in serum following the specifics of protocols previously described (for details please see [47, 48], for antibody concentrations please see Supplementary Table 4). Two animals were excluded from the final data analysis (one from the GH and one from the IST group). The earlier due to blood collection and processing issues (it was also a significant outlier) and the latter for being under the detection limit of the assay.

### Statistics

Normality was tested using the Kolmogorov-Smirnov test. Once normality was found, data were analyzed using a two-tailed Student’s *t* test, Pearson’s correlations, and Two-way ANOVAs. Once normality was not reached, Mann–Whitney U-test, Wilcoxon matched pairs W test, and Spearman correlations were performed. Agonist experiments used within-subjects design whereas antagonist, endogenous measurements, and ablation used between-subjects. For detailed statistics please see Supplementary Tables 1,2, 3, 5.

## Results

### Social isolation and aggression training exacerbate aggression in male mice and hyperactivate the HPG axis

IST mice spent more time displaying aggressive behavior compared to GH controls which was reflected in a higher percentage of time displaying aggression, threatening and attacking the intruder as well as a higher number of attacks and decreased latency to attack (Fig. 1A). Also, all IST animals displayed attacks whereas only 16% of the GH mice attacked the intruder (Fig. 1A, for detailed behavior please see Supplementary Table 1).

Regarding neuronal activity, IST animals showed increased colocalization of c-FOS and GnRH in the MS (Fig. 1B) and rPOA (Fig. 1C). Particularly MS-GnRH neuronal activity positively correlated with the percentage of time displaying aggression (Supplementary Table 5). AVPV-kisspeptin neurons, on the other hand, showed very low levels of co-localization with c-FOS. Nevertheless, IST animals exhibited a higher number of kisspeptin neurons in the AVPV compared to GH controls (Fig. 1D). Finally, KNDy neurons (NK3R-positive) in the ARC were also found to present higher colocalization with c-FOS, which positively correlated with aggression (Fig. 1E, Supplementary Table 5).

Finally, as NKB signaling in hypothalamic regions has been associated with enhanced aggression in isolated animals [39], we evaluated NK3R-colocalization in the dorsomedial (DMH) and lateral hypothalamus (LHp), regions known to be involved in the display of aggressive behavior [39, 49]. NK3R-positive neurons in both regions showed an increased colocalization with c-FOS in IST mice compared to GH controls (Supplementary Fig. 1). In addition, the percentage of time spent on aggressive behavior positively correlated with the percentage of colocalization in both regions (Supplementary Table 5).

**Fig. 2:**
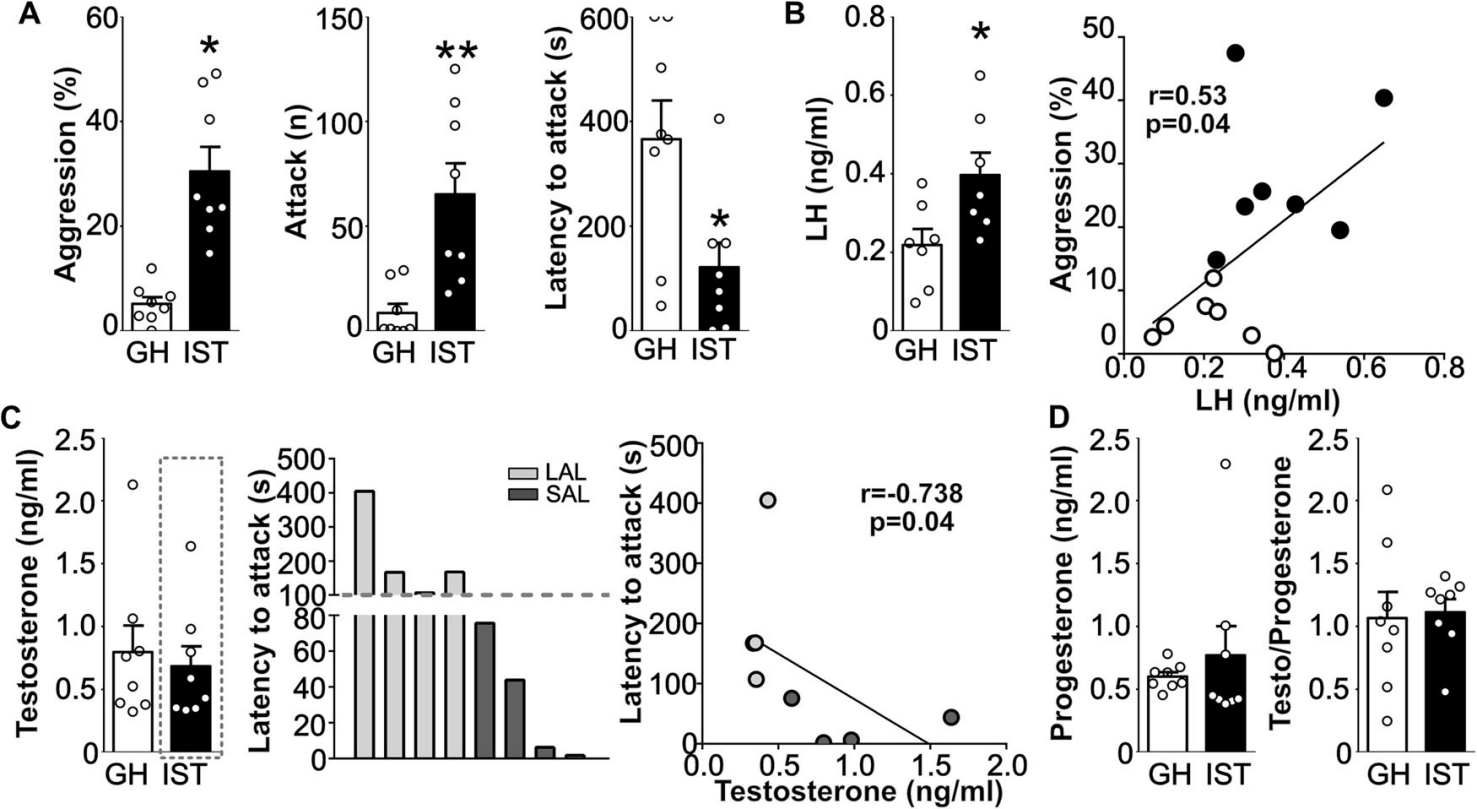
Highly aggressive IST mice exhibit elevated  LH levels in response to an aggressive encounter. Isolated and trained (IST, black bars) male mice spent a higher percentage of time on aggression (two-tailed Student’s *t* test t_(14)_ = 5.2, *p* = 0.0001), exhibited a higher number of attacks (Mann–Whitney U test U = 3.6, *p* = 0.002), and had a shorter attack latency (t_(14)_ = 2.8, *p* = 0.01) when compared to GH controls (white bars) (**A**). Regarding hormonal levels, IST mice exhibited higher levels of LH in serum (t_(14)_ = 2.5, *p* = 0.02), which positively correlated with aggression (Pearson’s correlation r = 0.53, *p* = 0.04) (**B**). Testosterone levels remained unchanged in IST animals. However, IST mice with a short attack latency (SAL, under 100 s) were found to present higher testosterone compared to long attack latency mice (LAL, over 100 s) (Latency to attack: U = 0, *p* = 0.02, Testosterone: U = 0, *p* = 0.02; Spearman’s correlation r = −0.73, *p* = 0.04) (**C**). Finally, progesterone and testosterone/progesterone ratio remained unchanged in IST animals compared to controls. *p < 0.05, **<0.01 vs GH (**D**). Data are presented as mean + s.e.m.

As the hypothalamic hubs regulating the HPG axis were found to be either highly active (GnRH and KNDy neurons, Fig. 1) or showed increased expression of peptide (AVPV-kisspeptin, Fig. 1C), we evaluated whether the IST protocol impacts the hormonal outputs of those peptides in a second cohort of animals. Once again IST mice exhibited higher levels of aggressive behavior reflected in a higher percentage of time in total aggression, threatening and attacking the intruder as well as a higher number of attacks and a reduced latency to attack (Fig. 2A, Supplementary Table 1).

Concerning serum hormonal levels, LH was found to be elevated in IST mice compared to low-aggressive GH controls. Of note LH concentrations positively correlated with the percentage of time displaying aggression (Fig. 2B, Supplementary Table 5). Interestingly, neither progesterone nor testosterone levels were found to be changed in IST mice or correlate with aggressive behavior when both groups were pooled (Fig. 2C, D, Supplementary Table 5). However, within the IST group, we found out that animals with a shorter attack latency (SAL, under 100 s) showed higher levels of testosterone in serum compared with animals with a higher attack latency (over 100 s) (Fig. 2C).

Altogether, these data indicate that isolation and aggression training lead to a hyperfunction of the HPG axis at the hypothalamic and pituitary levels.

### NK3R signaling mildly affects aggression in IST mice

As activation of NK3R and expression of NKB have been associated with escalated aggression in isolated mice, in the next set of experiments, we assessed whether agonism and antagonism of NK3Rs in IST males would affect aggressive behavior [39]. Pilot data (not shown) of our lab has demonstrated that senktide at the dose of 2 μg/Kg [39] had strong motor effects (crawling, tail rattle followed by immobility) 20 min after injection, thus we decided to use a lower dose of 1 μg/Kg and test behavior after 30 min.

**Fig. 3:**
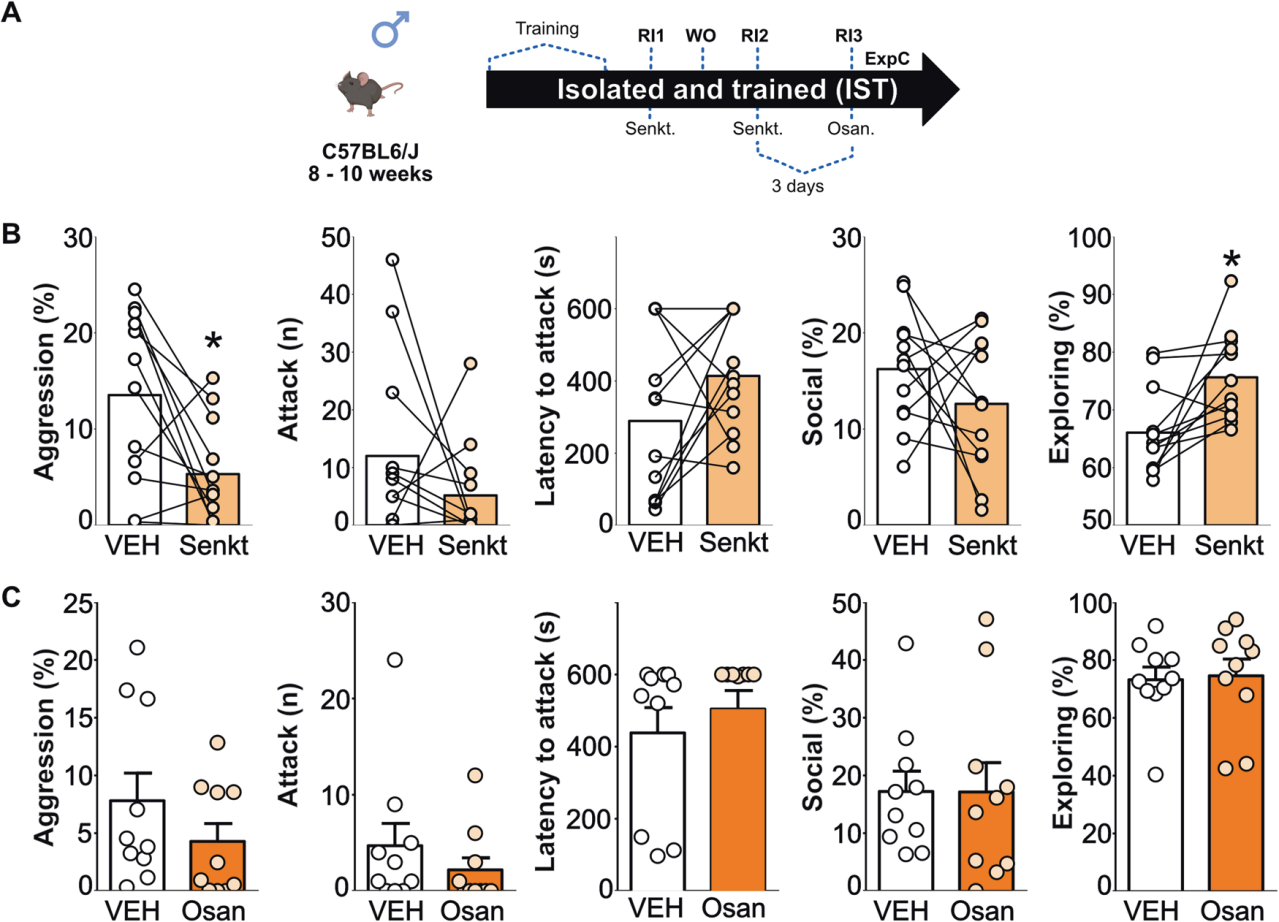
NK3R signaling mildly affects aggression in IST mice. Schematic drawings depicting the detailed protocols followed in experiments (Exp) C. Please note that each individual Exp has used different cohorts of animals. Osan: Osanetant (5 μg/Kg); RI: resident-intruder; Senkt: senktide (NK3R agonist), 1 μg/kg WO: Washout (neither behavior nor injection) (**A**). Senktide (light orange bars) reduced the total percentage of time spent on aggression (two-tailed Student’s *t* test t_(11)_ = 2.96, *p* = 0.01) and increased percentage of time spent exploring the homecage (t_(11)_ = 3.1, *p* = 0.01) but it did not affect the number of attacks, attack latency, and social investigation in IST mice (**B**). Osanetant (dark orange bars) did not affect the total percentage of time spent on aggression, the number of attacks, the latency to attack, or social investigation or homecage exploratory behaviors (**C**). *p < 0.05 vs vehicle. Data are presented as mean + s.e.m.

Our data showed that senktide decreased the percentage of time that IST mice spent on aggressive behavior which is mainly reflected by the reduced percentage of time spent on threats without significant changes in the number of attacks and latency to attack (Fig. 3B, Supplementary Table 2). Additionally, those animals seem to compensate for decreased time spent on aggression by increasing the percentage of time exploring their homecage. Importantly there were no motor effects at the 1 μg/Kg dose during testing (Supplementary Table 2).

Contrasting with previous data [39], the blockade of NK3Rs via osanetant did not affect any of the behaviors (Fig. 3C, Supplementary Table 2).

### High aggression in IST mice is underlied by GnRHR signaling

**Fig. 4:**
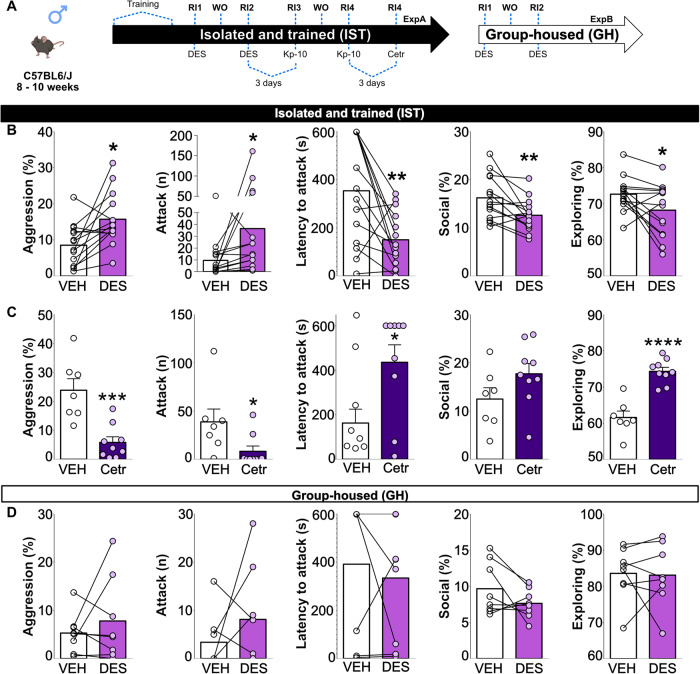
Exaggerated aggression in IST mice is underlied by GnRHR signaling. Schematic drawings depicting the detailed protocols followed in experiments (Exp) A and B. Please note that each individual Exp has used different cohorts of animals. Deso: deslorelin (GnRHR agonist, 300 ng/Kg); Kp-10: Kisspeptin-10 (0.52 μg/Kg); RI: resident-intruder; WO: Washout (neither behavior nor injection) (**A**). Deslorelin (Deso, GnRHR agonist, 300 ng/Kg, light purple bars) increased the total percentage of time spent on aggression (two-tailed Student’s *t* test t_(13)_ = 2.98, *p* = 0.01) as well as the number of attacks (Wilcoxon test W = 82, *p* = 0.007), and reduced attack latency (*t* test t_(13)_ = 3.39, *p* = 0.004), non-aggressive social investigations (*t* test t_(13)_ = 3.24, *p* = 0.006) and home cage exploration (*t* test t_(13)_ = 2.4, *p* = 0.03) in IST mice (**B**). Cetrorelix (Ctrx, GnRHR antagonist, 0.5 mg/Kg, dark purple bars) strongly reduced the total percentage of time spent on aggression (*t* test t_(6)_ = 6.36, *p* = 0.0007), the number of attacks (Mann–Whitney U test U = 10, *p* = 0.02), and increased the latency to attack U test (U = 13, *p* = 0.05) and homecage exploration (*t* test t_(14)_ = 0.79, *p* > 0.0001) in IST mice without affecting social investigation (**C**). Finally, deslorelin did not affect either aggressive social, or exploratory behaviors in group-housed (GH) mice (**D**). *p < 0.05, **<0.01 vs vehicle. Data are presented as mean + s.e.m.

As GnRH neuron activity (Fig. 1) and LH levels (Fig. 2) were increased in IST mice, we investigated whether manipulating GnRHR signaling via pharmacology affected aggressive behavior in IST mice (Fig. 4A).

Deslorelin (GnRHR agonist) exacerbated aggression in IST mice, those animals spent more time attacking and threatening as well as exhibited a higher number of attacks and decreased latency to attack (Fig. 4B, Supplementary Table 2). Additionally, GnRHR agonism reduced the percentage of time IST mice spend in non-aggressive social investigation (Fig. 4B, Supplementary Table 2). Consistently, blockade of GnRHRs via cetrorelix strongly reduced the time IST animals spend on aggression which was reflected in decreased time attacking, threatening, and decreased number of attacks and higher attack latency (Fig. 4C, Supplementary Table 2). Interestingly, the percentage of time spent on the social investigation was not affected by cetrorelix treatment.

As GnRHR activation led to exacerbated aggressive behavior in IST mice we wondered whether deslorelin would also increase aggression in low-aggressive GH mice. Surprisingly, there was no effect of deslorelin on GH mice’s aggressive or social behaviors (Fig. 4D, Supplementary Table 2).

### Kisspeptin and AVPV-Kisspeptin neurons differentially affect aggressive behavior induced by IST

As we have found (i) increased activation of KNDy neurons and (ii) a higher number of kisspeptin neurons in the AVPV of IST mice we decided to investigate the role of kisspeptin on aggression using behavioral pharmacology and loss of function experiments.

**Fig. 5:**
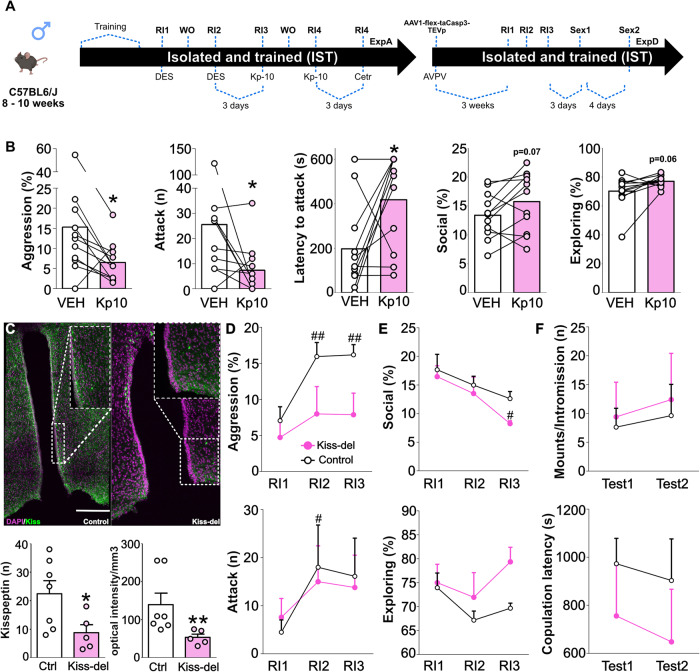
Kisspeptin and AVPV-Kisspeptin neurons differentially affect aggressive behavior in IST mice. Schematic drawings depicting the detailed protocols followed in experiments (Exp) A and D. Please note that each individual Exp has used different cohorts of animals. AVPV: anteroventral periventricular nucleus; Cetr: Cetrorelix (GnRHR antagonist, 0.5 mg/Kg); Deso: deslorelin (GnRHR agonist, 300 ng/Kg); Kp-10: Kisspeptin-10 (0.52 μg/Kg); RI: resident-intruder; Sex: sexual behavior test; WO: Washout (neither behavior nor injection) (**A**) Kisspeptin (Kp-10, 0.52 μg/Kg, pink bars) reduced the total percentage of time spent on aggression (two-tailed Student’s *t* test t_(10)_ = 2.51, *p* = 0.04) as well as the number of attacks (Wilcoxon test W = −39, *p* = 0.04), increased the attack latency (t_(10)_ = 2.53, *p* = 0.02), tended to elevate time spent on social investigation (t_(10)_ = 2.05, *p* = 0.06) and home cage exploration (t test t_(10)_ = 2.13, *p* = 0.06) in IST mice (**B**). Maximal z-projection of DAPI (magenta) and kisspeptin (green, Alexa-fluor 488 anti-rabbit) in the anteroventral periventricular nucleus (AVPV) confirming viral deletion of kisspeptin neurons (t_(10)_ = 2.29, *p* = 0.02) and reduction on fiber densities (Mann–Whitney U test U = 1, *p* = 0.005) (**C**). Finally, the deletion of kisspeptin neurons in the AVPV (Kiss-del, pink) abolished the rise in aggression levels seen in controls regarding the percentage of time spent on aggression (Two-way ANOVA Training effect: F_(2,22)_ = 20.10, *p* < 0.0001; Deletion effect: F_(1,11)_ = 4.16, *p* = 0.06; TrainingxDeletion: F_(2,22)_ = 4.55, *p* = 0.02) and number of attacks (Training effect: F_(2,22)_ = 3.93, *p* = 0.03; Deletion effect: F_(1,11)_ = 0.006, *p* = 0.93; TrainingxDeletion: F_(2,22)_ = 0.34, *p* = 0.77) (**D**), without affecting social, exploratory (**E**) and sexual behavior (**F**). *p < 0.05, **<0.01 vs vehicle or control (Ctrl); #<0.5 vs RI1; ## <0.1 vs RI1. Data are presented as mean + s.e.m. Scale bar 100 μm.

Surprisingly, contrasting with the effects of GnRH, kisspeptin strongly reduced aggression in IST mice. Those animals showed a decreased percentage of time spent on aggressive behavior, threat, and attacking as well as a decreased number of attacks and higher latency to attack (Fig. 5B, Supplementary Table 3). Furthermore, kisspeptin administration tended to increase time spent on social investigation (Fig. 5B) and increased home cage exploration (Supplementary Table 3).

Those pharmacological effects somewhat differed from the effect of ablation of AVPV kisspeptin neurons on aggression. Kiss::cre mice injected with the AAV1-TEV-Casp-3 virus showed a lower number of kisspeptin neurons as well as lower optical density /mm^2^ in the AVPV (Fig. 5C). According to the finding that IST animals showed a higher number of AVPV-kisspeptin neurons, ablation of those neurons suppressed the rise in time spent on aggression and number of attacks induced by aggression training without affecting baseline (first RI) levels of aggression (Fig. 5D, Supplementary Table 3). Moreover, those effects of AVPV-kisspeptin deletion seem to be specific to aggressive behavior as neuronal deletion did not affect male social, exploratory or sexual behavior (Fig. 5F) nor physiological measures such as testis relative weight (Supplementary Table 3).

## Discussion

We have shown here that the IST has a robust effect in enhancing aggression in male mice, similar to its effects in female rats [12, 45]. In addition, IST animals presented a hyperfunction of the HPG-axis, reflected by increased activity of GnRH and KNDy neurons and a greater number of AVPV-Kisspeptin neurons (Fig. 1). In line with our findings, hyperfunction of the HPG axis peptides has been associated with aggression in vertebrates. In fish, increased GnRH mRNA, particularly in the POA (where GnRH neurons were highly active in IST mice), has been associated with aggressive and dominant behavior [50, 51].

Consistently with exacerbated GnRH neuron activity, IST animals exhibited elevated serum LH levels (Fig. 2). These data are in line with previous observations in male mice using indirect methods of detection [29]. In this particular study, socially isolated mice that underwent multiple (4 to 16) aggressive interactions, showed elevated plasma LH levels. Furthermore, in our study, LH levels positively correlated with aggression indicating that high levels of LH (and GnRH activity[24]) might underlie heightened aggressive behavior. In fact, it has been demonstrated [29] that in violent sexual offenders high hostility and violent behavior are positively correlated with LH. Interestingly LH also correlated with recidivism which was not the case for testosterone levels, indicating that LH might be a better predictor of hostility and recidivism (re-engaging in aggression/aggression seeking) than testosterone.

Our data on GnRH neurons and LH support a model where hyperactivation of those neurons seen by colocalization with c-FOS and high serum LH leads to escalated aggression. This hypothesis was further confirmed by the finding that deslorelin (GnRHR agonist) enhanced whereas cetrorelix (GnRHR antagonist) decreased aggression in IST animals (Fig. 4). Interestingly, seminal studies in pigs and macaques have used GnRH agonists chronically to suppress HPG axis activity, reducing testosterone and consequently aggression [30, 31]. Those studies have ignored the fact that GnRH itself might affect behavior by acting in different brain regions independently or in synergism with testosterone. In fact, GnRHRs are widespread in the mouse brain [33], including in brain regions involved in aggressive behavior such as the VMH [52], LS [12] and CoA [53]. Further evidence of an independent role of GnRH neurotransmission on aggression comes from the finding that chronic administration of deslorelin exacerbates aggression and decreases social interactions in female Southern Giant Pouched rats (similarly to our acute effects in IST male mice) without affecting vaginal patency [54]. Additionally, blockade of GnRHRs was associated with decreased maternal aggression in lactating rats [55], another model where testosterone is not particularly involved.

Accordingly, compared to GH controls, IST animals showed no difference in testosterone levels in serum. LH and testosterone release are strongly linked [8, 14, 27, 56], so to some extent those results are surprising. Nevertheless, the lack of rise of testosterone might be due to the timepoint of blood collection (around 15 min after the RI), since previous studies in rats have shown that serum testosterone rises 1 h after an aggressive encounter [28]. Interestingly, we found that IST animals with SAL ( < 100 s) exhibit higher levels of testosterone in serum, which is consistent with testosterone being needed to “prime” the neuronal circuits of aggression to facilitate winning [8, 17, 57, 58]. However, whether this rise in testosterone levels reflects increased activity of GnRH neurons as seen in our model, remains to be elucidated. Nevertheless, future studies should assess whether the effects of GnRH signaling depend on testosterone levels and vice-versa.

In agreement with high GnRH neuron activity, KNDy neurons were found to be highly active in IST mice (Fig. 1) [36, 59, 60]. Curiously, a higher number of AVPV-kisspeptin neurons was found in IST mice (Fig. 1). Ablation of those neurons in Kiss::cre mice before the IST protocol abolished the rise in aggressive behavior induced via training without affecting baseline aggression (first RI) or sexual behavior (Fig. 5). Those effects (i) indicate a sex-specific role of AVPV-kisspeptin neurons on innate behaviors, i.e. involved in aggressive but not sexual behavior in males and sexual behavior in females [46] and (ii) highlight kisspeptin as a potential factor underlying the aggression training effect. Social isolation seems to strongly affect peptides as isolated rodents were found to present alterations related to peptide mRNA, synthesis, and release in the NKB [39], oxytocin [12, 13, 61], and vasopressin [12, 61] systems. As testosterone (and potentially aromatized estrogens) are known to induce plasticity in the aggression circuits of trained animals [8, 14], one could hypothesize that the increased number of kisspeptin neurons might arise from the plastic effects of estrogens or testosterone in kisspeptin synthesis and/or production in the AVPV. In fact, aromatase knockout (ArKO) male mice exhibited an increased number of kisspeptin neurons in the AVPV which were further heightened after castration and treatment with estradiol for 10 days [62, 63]. Additionally, testosterone treatment itself increased the promptness for aggression (reduced attack latency) in Peromyscus, similar to our training effect, whereas aromatase inhibition affected baseline aggression [58]. Taking together, one could hypothesize that aggressive experiences and social isolation alter different sex steroid sources to act on AVPV-kisspeptin neurons enhancing synthesis and release in order to generate the training-induced rise in aggression. Future studies should dissect the link between this IST-induced increase in the number of AVPV-kisspeptin neurons, sex steroids, and aggression.

Contrasting with the above-described results, administration of kisspeptin-10 i.p. strongly reduced aggression in males (Fig. 5). This result was somewhat puzzling and unexpected. However, one could hypothesize that (i) kisspeptin acts via other neuromodulatory systems to decrease aggression independently of GnRH or (ii) other atypical kisspeptin neuronal populations are responsible for these anti-aggressive effects. Regarding the first hypothesis, kisspeptin was found to act via nitric oxide (NO) and neuronal nitric oxide synthase (nNOS) to stimulate sexual behavior in females [24, 46, 64]. In males, decreased NO signaling is known to exacerbate aggression [65–68], thus one could hypothesize that kisspeptin decreases aggression in males by stimulating NO production and neurotransmission.

Concerning other kisspeptin neuronal populations, extrahypothalamic populations have been described in the LS and medial amygdala [69]. As both regions are known to send GABAergic projections to the VMH [53, 70, 71] to suppress aggressive behavior, one could hypothesize that those neurons are also the ones expressing kisspeptin. Future studies should dissect the neural circuits underlying the anti-aggressive effects of kisspeptin using site-specific gain and loss of function approaches.

To the best of our knowledge, this is the first study to assess the role of different levels of the hypothalamus-pituitary-gonad axis on aggressive behavior. Here we provided evidence indicating that escalated aggression is underlied by elevated activity of the GnRH system including high neuronal activity and, particularly, increased GnRHR signaling. Additionally, we described a novel role, beyond the reproductive context, for AVPV-kisspeptin neurons as well as kisspeptin signaling.

One could assume that social isolation and aggressive experiences create an imbalance in neuronal inhibitory inputs to the HPG axis leading to a hyperfunction of this system. This hyperfunctioning system probably generates exaggerated aggressive behavior seen in our animals. Interestingly aggressive interactions themselves might contribute to maintaining an “allostatic” and hyperfunctioning HPG axis by elevating levels of sex steroids such as testosterone which feedback directly or indirectly on kisspeptin and GnRH neurons. It remains to be addressed whether those behavioral and neuroendocrine alterations are also found in female mice. It has been shown that gonadectomy reduced aggression in female mice and rats [44, 72, 73], whereas chronic treatment with deslorelin was found to enhance aggression in female Southern Giant Pouched rats [54]. Furthermore, administration of a GnRHR antagonist decreased maternal aggression [55]. These findings thus suggest that the HPG axis is involved in female aggression. Indeed, preliminary unpublished data from our lab shows that IST females also presented an enhanced activity of MS GnRH and KNDy neurons when compared to GH controls. Therefore, future studies should investigate how the IST protocol affects the HPG axis of females.

Taken together, this study might impact the fields of neuroendocrinology and behavioral neuroscience opening new venues for the role of reproductive-relevant peptides in pathological behavioral conditions involving exacerbated aggression.

### Supplementary information

Supplementary information
