# Supplementary material for: Social isolation and aggression training lead to escalated aggression and hypothalamus-pituitary-gonad axis hyperfunction in mice
Source: Neuropsychopharmacology. 2024 Feb 9;49(8):1266–75. doi: 10.1038/s41386-024-01808-3 (PMC11224373; doi:10.1038/s41386-024-01808-3)
Supplement: Supplementary file 1 — Supplementary information [file 41386_2024_1808_MOESM1_ESM.docx]

**Supplementary info**

- 1. **Sexual behavior**

Sexual behavior was videotaped and took place in the early hours of the dark phase under dim red light conditions. Typically, a receptive (estrus) naturally cycling C57BL6/J mouse was introduced into the male’s home cage and left to interact freely with the experimental male for 20 minutes. Behavior was scored live using a pen, paper sheets, and stop-watch. Latencies for the first mount and first intromission as well as frequencies of mounts and intromissions were scored. The female estrous cycle was monitored via vaginal smears and histology. Additionally, receptivity was confirmed prior to the experiment by pairing the estrous females with stimulus males.

- 1. **Stereotaxic surgery**

Stereotaxic surgery was performed under semi-sterile conditions (1). Briefly, Kiss:cre or WT male mice were anesthetized with a mixture of isoflurane (3-4% for initial anesthesia followed by 1-2% for sustained anesthesia), injected i.p. with the analgesic Temgesic (0.05 mg/kg Buprenorphine) and fixed in a stereotaxic frame. Then, a small incision was made on the skin to expose the skull. Bregma was used as a landmark to find the AVPV (AP: 0.3; ML: ±0.1; DV: +5.4). After drilling the skull surface, animals were infused with 1µl of virus per side (0.1µl/min). Following injection, the injector was held in place for at least 10 min to allow viral diffusion. After the skin was sutured, mice were left to rest in their homecage until they were fully awake. The animal’s health status was monitored periodically for the next 2 days, and behavioral tests started 3 weeks after surgery.

- 1. **Drugs**

Deslorelin ([des-Gly10, D-Ala6]-LH-RH ethylamide acetate salt hydrate, L4513, Sigma-Aldrich) was chosen to be a selective and potent GnRH-R agonist (2). Furthermore, the dose of 300ng/Kg was shown to activate LH release (3). Cetrorelix acetate (0.5mg/Kg, C5249, Sigma-Aldrich, diluted in 1% methanol) was chosen to be a potent inhibitor of the HPG axis and LH release (4). Kisspeptin 10 (Kp-10) was injected subcutaneously at the dose of 0.52μg/Kg as this dose was previously described to stimulate female sexual behavior in our lab (1,5). Senktide and osanetant NK3R receptor agonist and antagonist were injected in doses of 1μg/Kg and 5μg/Kg, respectively. These doses were shown to influence aggressive behavior in socially isolated mice (6). Importantly, we conducted pilot experiments in GH and IST animals using higher doses of senktide (2μg/Kg, 20min prior experiment) as previously described (34), however using this dose, induced strong motor effects such as crawling, immobility, and tail rattling (data not shown).

- 1. **Immunohistochemistry**

After perfusion, brains were cryo-cutted and slices (40µm) containing the target regions were collected in cryoprotectant solution and stored at -20°C until the experiment took place. A series of 6-8 slices were used for immunostaining. For neuronal activity, slices were washed in 0.1 PBS, and incubated in a solution of 3% H_2_O_2_ and 10% methanol for 15 min at room temperature (RT). Afterwards, slices were washed with PBS 0.1 M and blocked for 1 hour in blocking solution (Normal goat serum, NGS 5% 0.1 PBS with 0.3% triton-x 100). Directly after blocking, slices were incubated in primary antibody anti-cFOS antibody at 4°C for 48h. Next, brain sections were washed in PBS 0.1 M, and incubated with the secondary antibody. After incubation, slices were washed in PBS, incubated for 1 hour in the avidin-biotin (ABC kit, PK-4000, Vector Labs), washed in PBS, and incubated for 5 min in a gray substrate for peroxidase solution (SK-4700, Vector labs). Slices were then washed in PBS and incubated in the blocking solutions to block avidin and biotin (SP-2001, vector labs) after another round of washes in PBS. Slices were then incubated for 48 hours (4°C) with primary antibodies (NGS 1% 0.1 PBS with 0.3% triton-x 100) targeting the peptides of interest (for details of blocking and primary antibodies please see Supplementary Table 4). Then, slices followed similar steps as the ones described above, however, they were incubated in a violet peroxidase substrate (SK-4600, Vector labs). Finally, slices were rinsed in 0.1 PBS and mounted on adhesive microscope slides (Superfrost Plus, Thermo Fisher Scientific), left to dry overnight, dehydrated in xylene, and mounted in Eukit (03989, Sigma, Aldrich).

For immunofluorescence, brain slices were washed in 0.1 PBS, incubated in a solution of 3% H2O2 and 10% methanol for 15 min at room temperature (RT), then rinsed in Glycine buffer (0.1M in PBS) for 20 min. Afterwards, slices were washed with PBS and blocked for 1 hour in a solution of normal goat or (NGS 5% 0.1 PBS with 0.3% triton-x 100). Directly after blocking, slices were incubated in primary antibody rabbit-anti-kisspeptin-10 antibody (1:1000, #AC566, Caraty antibody, INRAE, Tours, France) at 4°C for 48h. Next, slices were washed in PBS and incubated for 2 hours in RT with a solution of DAPI (1:10000) and a secondary antibody (Alexa-fluor 488 goat-anti-rabbit, 1:500, Thermofisher). After secondary antibody incubation, brain sections were rinsed in 0.1 PBS and mounted (Aqua-Poly mount medium, #18606-20) on adhesive microscope slides (Superfrost Plus, Thermo Fisher Scientific Inc, USA). Slides were kept in the dark at 4°C until imaging.

Florescence slides were processed using an inverted confocal laser scanning microscope (Leica SP5, Leica Microsystems, GIGA-Cell imaging platform). Immunoperoxidase slides were imaged using an Epifluorescence Microscope (Echo Revolve, GIGA-Cell imaging Platform). Digital images were processed (Merging and Z-projections) using the Leica Application Suite X (Leica) and Fiji70. Cell counting was done by an experienced observer blind to the treatments (7).


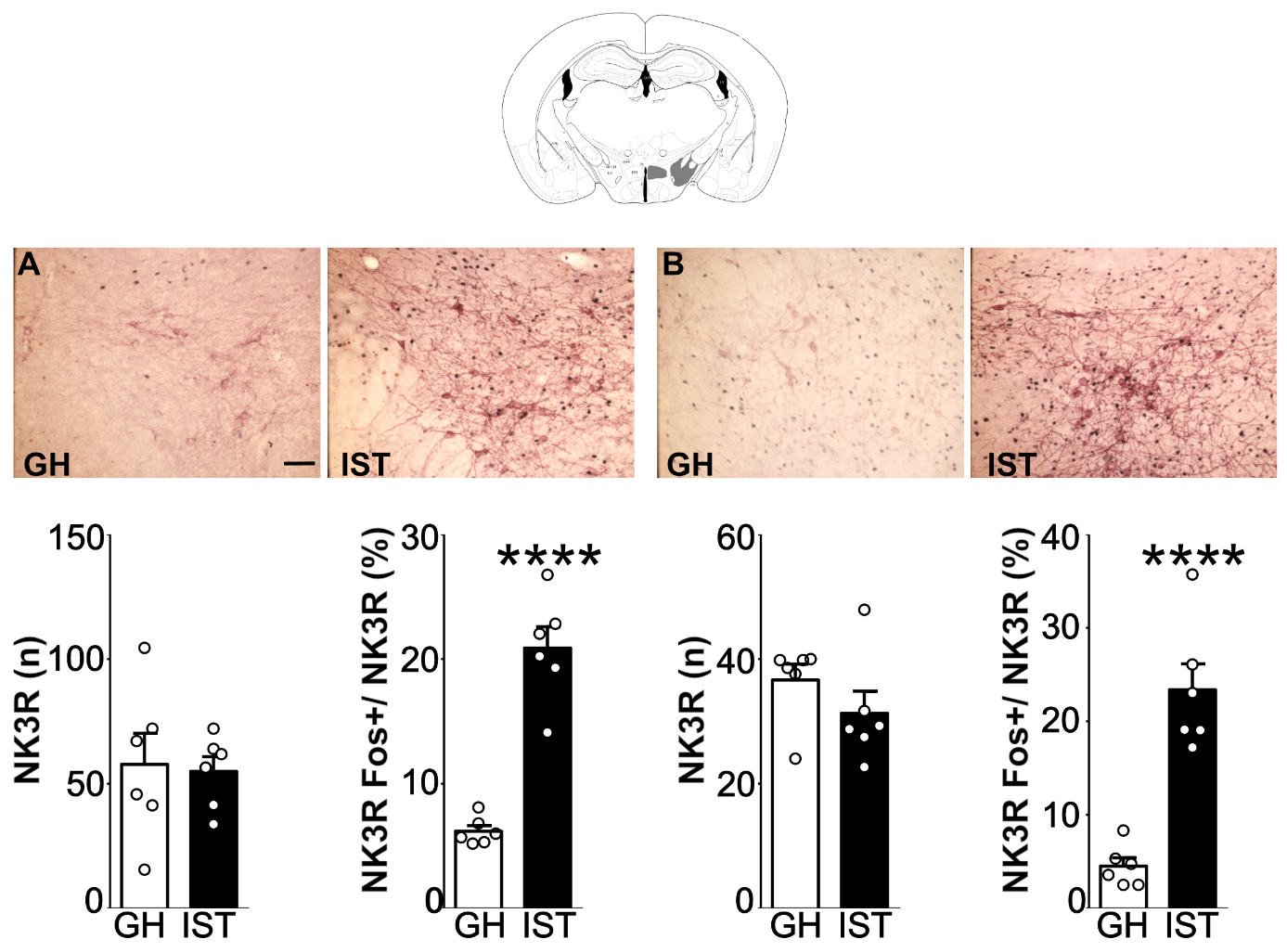


**Supplementary figure 1**. Isolated and trained mice (IST, black bars) exhibited a higher percentage of NK3R-positive neurons co-expressing cFOS in the dorsomedial (t_(10)_= 8.3, p<0.0001) (A) and lateral hypothalamus (t_(10)_= 6.4, p<0.0001) (B) when compared to group-housed (white bars) animals. Total neuronal numbers expressing NK3R did not differ in both regions independent of the groups (LH: U=10, p=0.22; DMH: t_(10)_= 0.2, p=0.84. ****p<.0001 vs GH. Data are presented as mean + s.e.m. Scale bar 100μm.

**Supplementary Table 1:** Overview of detailed statistical analysis as well as p values for behavioral data displayed in Figures 1 and 2. Significant p values are in bold.

| **Figure** | **Data** | **Detailed Statistics** | **pvalue** | **Numbers** |
| --- | --- | --- | --- | --- |
| **1** | Aggression | two-tailed Student’s t-test t_(10)_= 2.93 | **0.01** | N=6 |
|  | Attack number | Mann-Whitney U test U= 3.0 | **0.01** |  |
|  | Attack latency | Mann-Whitney U test U= 0.0 | **0.002** |  |
|  | Attack time (%) | Mann-Whitney U test U= 4.0 | **0.02** |  |
|  | Threat (%) | Mann-Whitney U test U= 0.0 | **0.002** |  |
|  | Social (%) | two-tailed Student’s t-test t_(10)_=0.56 | 0.584 |  |
|  | Exploring (%) | two-tailed Student’s t-test t_(10)_= 2.97 | **0.01** |  |
|  | Grooming (%) | two-tailed Student’s t-test t_(10)_= 2.97 | 0.177 |  |
| **2** | Aggression | two-tailed Student’s t-test t_(14)_= 5.21 | **0.0001** | N=8 |
|  | Attack number | Mann-Whitney U test U= 4.0 | **0.001** |  |
|  | Attack latency | two-tailed Student’s t-test t_(14)_= 2.8 | **0.013** |  |
|  | Attack time (%) | Mann-Whitney U test U= 3.0 | **0.0003** |  |
|  | Threat (%) | Mann-Whitney U test U= 3.0 | **0.001** |  |
|  | Social (%) | two-tailed Student’s t-test t_(14)_=4.28 | **0.0008** |  |
|  | Exploring (%) | two-tailed Student’s t-test t_(14)_= 3.51 | **0.003** |  |
|  | Grooming (%) | two-tailed Student’s t-test t_(14)_= 3.16 | **0.006** |  |

**Supplementary Table 2:** Overview of detailed statistical analysis as well as p values for behavioral data displayed in Figure 3 and 4. Significant p values are in bold and nearly significant in italics. Cetr: Cetrorelix.

| **Figure** | **Data** | **Detailed Statistics** | **pvalue** | **Numbers** |
| --- | --- | --- | --- | --- |
| **3b** | Aggression | Paired, two-tailed Student’s t-test t_(11)_= 2.95 | **0.01** | N=12 |
|  | Attack number | Wilcoxon matched-pairs test W= -28 | 0.23 |  |
|  | Attack latency | Wilcoxon matched-pairs test W= 32 | 0.17 |  |
|  | Attack time (%) | Wilcoxon matched-pairs test W= 34 | 0.14 |  |
|  | Threat (%) | Paired, two-tailed Student’s t-test t_(11)_= 2.72 | **0.02** |  |
|  | Social (%) | Paired, two-tailed Student’s t-test t_(11)_= 1.18 | 0.26 |  |
|  | Exploring (%) | Paired, two-tailed Student’s t-test t_(11)_= 3.1 | **0.01** |  |
|  | Grooming (%) | Wilcoxon matched-pairs test W= 50 | *0.05* |  |
| **3c** | Aggression | two-tailed Student’s t-test t_(18)_= 1.23 | 0.23 | N=10 |
|  | Attack number | Mann-Whitney U test U= 35.5 | 0.26 |  |
|  | Attack latency | Mann-Whitney U test U= 33 | 0.19 |  |
|  | Attack time (%) | Mann-Whitney U test U= 36 | 0.29 |  |
|  | Threat (%) | Mann-Whitney U test U= 34 | 0.25 |  |
|  | Social (%) | two-tailed Student’s t-test t_(18)_=0.01 | 0.98 |  |
|  | Exploring (%) | two-tailed Student’s t-test t_(18)_= 0.18 | 0.85 |  |
|  | Grooming (%) | Mann-Whitney U test U= 46 | 0.78 |  |
| **4b** | Aggression | Paired, two-tailed Student’s t-test t_(13)_= 2.98 | **0.01** | N=14 |
|  | Attack number | Wilcoxon matched-pairs test W= 82 | **0.007** |  |
|  | Attack latency | Paired, two-tailed Student’s t-test t_(13)_= 3.39 | **0.004** |  |
|  | Attack time (%) | Paired, two-tailed Student’s t-test t_(13)_= 2.16 | **0.04** |  |
|  | Threat (%) | Paired, two-tailed Student’s t-test t_(13)_= 3.38 | **0.004** |  |
|  | Social (%) | Paired, two-tailed Student’s t-test t_(13)_= 3.25 | **0.006** |  |
|  | Exploring (%) | Paired, two-tailed Student’s t-test t_(13)_= 2.4 | **0.03** |  |
|  | Grooming (%) | Paired, two-tailed Student’s t-test t_(13)_= 1.06 | 0.30 |  |
| **4c** | Aggression | two-tailed Student’s t-test t_(14)_= 4.38 | **0.0006** | VEH=7; Cetr=9 |
|  | Attack number | Mann-Whitney U test U= 10 | **0.02** |  |
|  | Attack latency | two-tailed Student’s t-test t_(14)_= 2.59 | **0.02** |  |
|  | Attack time (%) | Mann-Whitney U test U= 7.0 | **0.007** |  |
|  | Threat (%) | two-tailed Student’s t-test t_(14)_= 3.89 | **0.001** |  |
|  | Social (%) | two-tailed Student’s t-test t_(14)_=1.63 | 0.12 |  |
|  | Exploring (%) | two-tailed Student’s t-test t_(14)_=6.28 | **<0.0001** |  |
|  | Grooming (%) | two-tailed Student’s t-test t_(14)_=0.79 | 0.44 |  |
| **4d** | Aggression | Paired, two-tailed Student’s t-test t_(7)_= 0.84 | 0.42 | N=8 |
|  | Attack number | Wilcoxon matched-pairs test W= 9 | 0.31 |  |
|  | Attack latency | Paired, two-tailed Student’s t-test t_(7)_= 0.67 | 0.52 |  |
|  | Attack time (%) | Wilcoxon matched-pairs test W= 7 | 0.44 |  |
|  | Threat (%) | Paired, two-tailed Student’s t-test t_(7)_= 0.98 | 0.35 |  |
|  | Social (%) | Paired, two-tailed Student’s t-test t_(7)_=1.65 | 0.14 |  |
|  | Exploring (%) | Paired, two-tailed Student’s t-test t_(7)_=0.16 | 0.87 |  |
|  | Grooming (%) | Wilcoxon matched-pairs test W= -4 | 0.84 |  |

**Supplementary Table 3:** Overview of detailed statistical analysis as well as p values for behavioral data displayed in Figure 5. Significant p values are in bold and nearly significant in italics.

| **Figure** | **Data** | **Detailed Statistics** | **pvalue** | **Numbers** |
| --- | --- | --- | --- | --- |
| **5b** | Aggression | Paired, two-tailed Student’s t-test t_(10)_= 2.52 | **0.03** | N=11 |
|  | Attack number | Wilcoxon matched-pairs test W= -39 | **0.04** |  |
|  | Attack latency | Paired, two-tailed Student’s t-test t_(10)_= 2.54 | **0.03** |  |
|  | Attack time (%) | Wilcoxon matched-pairs test W= -37 | *0.06* |  |
|  | Threat (%) | Paired, two-tailed Student’s t-test t_(10)_= 2.79 | **0.02** |  |
|  | Social (%) | Paired, two-tailed Student’s t-test t_(10)_= 2.05 | *0.07* |  |
|  | Exploring (%) | Paired, two-tailed Student’s t-test t_(10)_= 2.13 | *0.06* |  |
|  | Grooming (%) | Paired, two-tailed Student’s t-test t_(10)_= 0.56 | 0.59 |  |
| **5c** | Kisspeptin (n) | two-tailed Student’s t-test t_(10)_=2.29 | **0.04** | Ctrl= 8  Kiss-del=5 |
|  | Optical intensity/mm^2^ | Mann-Whitney U test U= 1.00 | **0.005** |  |
|  | Testis relative weight | two-tailed Student’s t-test t_(10)_=1.53  Ctrl= 0.01±0.0008 Kiss-del= 0.008±0.0007 | 0.15  (mean±SEM) |  |
| **5d-e** | 2 way ANOVA followed by Bonferroni’s multiple comparisons test | | |  |
|  | Aggression (%) | Virus effect: F_(1,11)_=4.16 | *0.06* |  |
|  |  | Training effect: F_(2,22)_=20.10 | **<0.0001** |  |
|  |  | Virus x training effect: F_(2,22)_=4.55 | **0.02** |  |
|  | Attack number | Virus effect: F_(1,11)_=0.006 | 0.93 |  |
|  |  | Training effect: F_(2,22)_=3.93 | **0.03** |  |
|  |  | Virus x training effect: F_(2,22)_=0.34 | 0.71 |  |
|  | Attack time (%) | Virus effect: F_(1,11)_=0.751 | 0.4 |  |
|  |  | Training effect: F_(2,22)_=5.660 | **0.01** |  |
|  |  | Virus x training effect: F_(2,22)_=0.237 | 0.79 |  |
|  | Attack latency | Virus effect: F_(1,11)_=0.45 | 0.51 |  |
|  |  | Training effect: F_(2,22)_=2.89 | *0.08* |  |
|  |  | Virus x training effect: F_(2,22)_=0.28 | 0.75 |  |
|  | Threat (%) | Virus effect: F_(1,11)_=4.39 | *0.06* |  |
|  |  | Training effect: F_(2,22)_=3.45 | **0.04** |  |
|  |  | Virus x training effect: F_(2,22)_=2.29 | 0.12 |  |
|  | Social (%) | Virus effect: F_(1,11)_=2.09 | 0.17 |  |
|  |  | Training effect: F_(2,22)_=4.98 | **0.02** |  |
|  |  | Virus x training effect: F_(2,22)_=0.34 | 0.56 |  |
|  | Exploring (%) | Virus effect: F_(1,11)_=3.03 | 0.11 |  |
|  |  | Training effect: F_(2,22)_=2.42 | 0.11 |  |
|  |  | Virus x training effect: F_(2,22)_=1.4 | 0.27 |  |
| **5f** | Mounts  Intromissions | Virus effect: F_(1,11)_=0.10 | 0.74 |  |
|  |  | Training effect: F_(1,11)_=0.43 | 0.52 |  |
|  |  | Virus x training effect: F_(1,11)_=0.02 | 0.89 |  |
|  | Copulation  Latency | Virus effect: F_(1,11)_=0.25 | 0.49 |  |
|  |  | Training effect: F_(1,11)_=0.50 | 0.49 |  |
|  |  | Virus x training effect: F_(1,11)_=0.0.04 | 0.83 |  |

**Supplementary Table 4:** Overview of antibodies for immunolabelling protocols

| **Immunohistochemistry peroxidase** | | | | | |
| --- | --- | --- | --- | --- | --- |
| **Target** | **First round of staining** | | | **Second round of staining** | |
|  | **Primary**  **antibody** | **Blocking solution** | **Secondary**  **Antibody** | **Primary**  **antibody** | **Secondary**  **Antibody** |
| *GnRH* | rabbit-anti-cFOS antibody (1:2500 abcam #ab190289) | NGS 5% | Biotin-SP AffiniPure-  Goat-Anti-Rabbit  (1:250; #111-065-003,  JacksonImmuno  Research) | Guinea-pig-anti-GnRH antibody (1:2000),  #1018  Eric Hrabovsky(4,8,11) | Biotin-SP AffiniPure-Goat Anti-Guinea Pig IgG Antibody (1:250#106-065-003,  JacksonImmuno  Research |
| *Kisspeptin* | guineapig-anti-cFOS antibody (1:1000 synapticsystems #226-005) |  | Biotin-SP AffiniPure-Goat Anti-Guinea Pig IgG Antibody (1:250# 106-065-003,  JacksonImmuno  Research | Rabbit-anti-kisspeptin (1:10000), #AC566, INRAE, Isabelle Franceschini(9,12) | Biotin-SP AffiniPure-  Goat-Anti-Rabbit  (1:250; #111-065-003,  JacksonImmuno  Research) |
| *NK3R* | rabbit-anti-cFOS antibody (1:2500 abcam #ab190289) |  | Biotin-SP AffiniPure-  Goat-Anti-Rabbit  (1:250; #111-065-003,  JacksonImmuno  Research) | Rabbit-anti-NK3R (1:2000), IS-7/7, Inserm, Bordeaux, Phillip Ciofi(10,13) | Biotin-SP AffiniPure-  Goat-Anti-Rabbit  (1:250; #111-065-003,  JacksonImmuno  Research) |
| **Immunofluorescence** | | | | | |
| *Kisspeptin* | Rabbit-anti-kisspeptin (1:1000), #AC566, INRAE, Isabelle Franceschini | NGS 5% | Alexa-fluor  488 goat-anti-  Rabbit (1:500) Thermofisher | - | |
| **LH assay** | | | | | |
| *Luteinizing*  *Hormone*  *(LH)* | Rabbit polyclonal-anti- LH antibody (1:10000)  AFP240580Rb  bovine LHβ 518B7 monoclonal Ab (1:1000) - mouse LH reference (AFP-5306A) 400 to 0,19pg/ml. | PBS-Tween20-Milkpowder (0.1M PBS, 0.05% Tween 20, 5% milkpowder) | DAKO Cytomation polyclonal Goat Anti-Rabbit (1:1000)  IgG/HRP cat# P0448. | - | |

**Supplementary Table 5:** Overview of correlation coefficients between aggressive behavior, displayed by group-housed and isolated and trained male mice (combined) during the RI, and the percentage of colocalization of c-Fos and peptide of interest or hormonal levels in serum. Significant correlations are in bold.

| **Peptide of interest-Fos (%)** | | | | |
| --- | --- | --- | --- | --- |
| **Peptide** | **r** | **pvalue** | **correlation** | **Figure** |
| MS GnRH | 0.6643 | **0.0214** | Spearman’s | **1** |
| rPOA GnRH | 0.5285 | 0.0977 |  |  |
| AVPV kisspeptin | 0.2357 | 0.4609 | Pearson’s |  |
| NK3R (ARC) | 0.7666 | **0.0036** |  |  |
| NK3R (DH | 0.6660 | **0.0180** |  |  |
| NK3R (LH) | 0.7259 | **0.0075** |  |  |
| **Hormonal measurements** | | | | |
| Hormone | **r** | **pvalue** | **correlation** | **Figure** |
| LH | 0.5361 | **0.0481** | Pearson’s | 2 |
| Testosterone | -0.2479 | 0.3546 |  |  |
| Progesterone | -0.05891 | 0.8284 |  |  |
| Testosterone (IST) | -0.7381 | **0.0458** | Spearman’s |  |

**References**

1. Hellier V, Brock O, Candlish M, Desroziers E, Aoki M, Mayer C, *et al.* (2018): Female sexual behavior in mice is controlled by kisspeptin neurons. *Nat Commun* 9. https://doi.org/10.1038/s41467-017-02797-2

2. Lo B, Freeman AR, Singh B, Hanadari-Levy A, Berman J, Chaves A, *et al.* (2022): Effects of a Gonadotropin-Releasing Hormone Agonist on Sex Behavior in Females of the Southern Giant Pouched Rat. *Integrative and Comparative Biology*, vol. 62 62: 613–624.

3. Tata B, Huijbregts L, Jacquier S, Csaba Z, Genin E, Meyer V, *et al.* (2014): Haploinsufficiency of Dmxl2, Encoding a Synaptic Protein, Causes Infertility Associated with a Loss of GnRH Neurons in Mouse. *PLoS Biol* 12. https://doi.org/10.1371/journal.pbio.1001952

4. Tata B, Mimouni NEH, Barbotin AL, Malone SA, Loyens A, Pigny P, *et al.* (2018): Elevated prenatal anti-Müllerian hormone reprograms the fetus and induces polycystic ovary syndrome in adulthood. *Nat Med* 24: 834–846.

5. Silva MSB, Decoster L, Trova S, Mimouni NEH, Delli V, Chachlaki K, *et al.* (2022): Female sexual behavior is disrupted in a preclinical mouse model of PCOS via an attenuated hypothalamic nitric oxide pathway. *Proc Natl Acad Sci U S A* 119: 1–12.

6. Zelikowsky M, Hui M, Karigo T, Choe A, Yang B, Blanco MR, *et al.* (2018): The Neuropeptide Tac2 Controls a Distributed Brain State Induced by Chronic Social Isolation Stress. *Cell* 173: 1265-1279.e19.

7. Schindelin J, Arganda-carreras I, Frise E, Kaynig V, Longair M, Pietzsch T, *et al.* (2012): Fiji : an open-source platform for biological-image analysis. *Nat Methods* 9: 676–682.

8. Kalló I, Vida B, Deli L, Molnár CS, Hrabovszky E, Caraty A, *et al.* (2012): Co-Localisation of Kisspeptin with Galanin or Neurokinin B in Afferents to Mouse GnRH Neurones. *J Neuroendocrinol* 24: 464–476.

9. Franceschini I, Lomet D, Cateau M, Delsol G, Tillet Y, Caraty A (2006): Kisspeptin immunoreactive cells of the ovine preoptic area and arcuate nucleus co-express estrogen receptor alpha. *Neurosci Lett* 401: 225–230.

10. Mittelman-Smith MA, Williams H, Krajewski-Hall SJ, Lai J, Ciofi P, McMullen NT, Rance NE (2012): Arcuate kisspeptin/neurokinin B/dynorphin (KNDy) neurons mediate the estrogen suppression of gonadotropin secretion and body weight. *Endocrinology* 153: 2800–2812.

11. Skrapits K, Kanti V, Savanyú Z, Maurnyi C, Szenci O, Horváth A, *et al.* (2015): Lateral hypothalamic orexin and melanin-concentrating hormone neurons provide direct input to gonadotropin-releasing hormone neurons in the human. *Front Cell Neurosci* 9. https://doi.org/10.3389/fncel.2015.00348

12. Clarkson J, d’Anglemont de Tassigny X, Colledge WH, Caraty A, Herbison AE (2009): Distribution of kisspeptin neurones in the adult female mouse brain. *J Neuroendocrinol* 21: 673–682.

13. Griffond B, Ciofi P, Bayer L, Jacquemard C, Fellmann D, Ura NRS (1997): Immunocytochemical detection of the neurokinin B receptor (NK3) on melanin-concentrating hormone (MCH) neurons in rat brain. *Journal of Chemical Neuroanatomy*, vol. 12.
